# Docosahexaenoic Acid Alleviates Trimethylamine-*N*-oxide-mediated Impairment of Neovascularization in Human Endothelial Progenitor Cells

**DOI:** 10.3390/nu15092190

**Published:** 2023-05-04

**Authors:** Jia-Ning Syu, Hung-Yu Lin, Tun Yu Huang, Der-Yen Lee, En-Pei Isabel Chiang, Feng-Yao Tang

**Affiliations:** 1 Biomedical Science Laboratory, Department of Nutrition, China Medical University, Taichung 40604, Taiwan; mine800815@gmail.com; 2 Research Assistant Center, Show Chwan Memorial Hospital, Changhua 500, Taiwan; linhungyu700218@gmail.com; 3 Prospective Wound Medicine Research Center, Show Chwan Memorial Hospital, Changhua 500, Taiwan; hdytll@gmail.com; 4 Graduate Institute of Integrated Medicine, China Medical University, Taichung 40604, Taiwan; deryen.lee@mail.cmu.edu.tw; 5 Department of Food Science and Biotechnology, National Chung Hsing University, Taichung 402, Taiwan; chiangisabel@email.nchu.edu.tw; 6 Innovation and Development Center of Sustainable Agriculture (IDCSA), National Chung Hsing University, Taichung 402, Taiwan

**Keywords:** trimethylamine-*N*-oxide, docosahexaenoic acid, endothelial nitric oxide synthase, microRNA 221, glutathione, endothelial progenitor cells

## Abstract

Background: Human endothelial progenitor cells (hEPCs), originating from hemangioblasts in bone marrow (BM), migrate into the blood circulation, differentiate into endothelial cells, and could act as an alternative tool for tissue regeneration. In addition, trimethylamine-*N*-oxide (TMAO), one of the gut microbiota metabolites, has been identified as an atherosclerosis risk factor. However, the deleterious effects of TMAO on the neovascularization of hEPCs have not been studied yet. Results: Our results demonstrated that TMAO dose-dependently impaired human stem cell factor (SCF)-mediated neovascularization in hEPCs. The action of TMAO was through the inactivation of Akt/endothelial nitric oxide synthase (eNOS), MAPK/ERK signaling pathways, and an upregulation of microRNA (miR)-221. Docosahexaenoic acid (DHA) could effectively inhibit the cellular miR-221 level and induce the phosphorylation level of Akt/eNOS, MAPK/ERK signaling molecules, and neovascularization in hEPCs. DHA enhanced cellular amounts of reduced form glutathione (GSH) through an increased expression of the gamma-glutamylcysteine synthetase (γ-GCS) protein. Conclusions: TMAO could significantly inhibit SCF-mediated neovascularization, in part in association with an upregulation of miR-221 level, inactivation of Akt/eNOS and MAPK/ERK cascades, suppression of γ-GCS protein, and decreased levels of GSH and GSH/GSSG ratio. Furthermore, the DHA could alleviate the detrimental effects of TMAO and induce neovasculogenesis through suppression of miR-221 level, activation of Akt/eNOS and MAPK/ERK signaling cascades, increased expression of γ-GCS protein, and increment of cellular GSH level and GSH/GSSG ratio in hEPCs.

## 1. Introduction

It is known that hEPCs mainly originate from BM and migrate to injured sites for neovascularization, the tubular formation of blood vessels, during tissue damage [1]. Those BM-originated hEPCs could develop into vascular lineage cells, including endothelial cells (ECs) and endothelial colony-forming cells (ECFCs), for tissue regeneration and repairment of vascular vessels in various pathological conditions [2]. The results from previous studies showed a strong positive association between circulating hEPCs and the prevention of atherosclerotic diseases such as myocardial infarction (MI), cardiovascular disease (CVD), and stroke [3,4]. The human stem cell factor (SCF) plays a central role in the induction of neovasculogenic functions and activities in hEPCs through the c-kit receptor protein. SCF-mediated activation of c-kit further leads to the activation of important signaling pathways, including Akt/endothelial nitric oxide synthase (eNOS) and mitogen-activated protein kinase (MAPK)/extracellular signal-regulated kinase (ERK) pathways [5]. Our previous findings also demonstrated that activation of eNOS by Akt would induce the migration and vascularization of hEPCs [6].

It is a well-known fact that dietary patterns and gut microbiota play important roles in determining the incidence of chronic diseases such as atherosclerosis and CVD [7,8,9]. Previous studies indicated that red meats are abundant in carnitine, choline, and betaine [10,11]. Dietary carnitine, choline, and betaine are metabolized into trimethylamine (TMA) in the intestine and subsequently turned into trimethylamine-*N*-oxide (TMAO) in the liver [12,13]. Several studies suggest that TMAO is associated with the incidence of diabetes, metabolic syndrome (MetS), and CVD [12,14]. A clinical study indicated that plasma levels of TMAO were negatively associated with blood-circulating hEPCs in human stable angina subjects [15]. These findings indicated that TMAO could impair the repair capability and function of hEPCs. A previous study indicated that TMAO induced inflammation through the augmentation of the nuclear factor-κB (NF-κB) signaling cascade in ECs [16].

In addition to signaling pathways, epigenetic regulators such as microRNAs (miRs) have become a subclass of noncoding RNAs that modulate the expression of targeted mRNAs [17]. A recent study indicated that microRNA-221 (miR-221) augmented the inflammatory response and injury during pathological conditions [18]. Our previous findings demonstrated that miR-221 is involved in the inactivation of Akt/eNOS signaling molecules and inhibition of neovasculogenesis in hEPCs [19]. A recent study suggested that plasma levels of miR-221 were negatively correlated with circulating EPC in CVD patients [20].

Previous studies showed that dietary intake of fish oil is associated with a lower incidence of inflammatory diseases and a decreased risk of CVD [21,22]. A recent study further indicated that consumption of fish is also correlated with lower plasma levels of TMAO in human subjects [23,24]. Our previous study demonstrated that DHA could prevent the dysfunction of hEPCs [6,25]. However, the inhibitory effects of TMAO on interfering SCF/c-kit and downstream Akt/eNOS signaling pathways have not been studied yet. Up-to-date, the beneficial effects of DHA on prevention of TMAO-mediated impairment of neovasculogenesis and inactivation of Akt/eNOS signaling pathways have not been well demonstrated yet. Therefore, we would investigate the mechanisms of action of TMAO in hEPCs. We will also examine the beneficial roles of DHA in the prevention of TMAO-modulated dysfunction and impairment of neovasculogenesis in hEPCs.

## 2. Materials and Methods

### 2.1. Chemical Reagents, Antibodies and Supplies

Trimethylamine *N*-oxide (TMAO), MCDB-131 medium, calcein-AM, and 3-[4,5-dimethhylthiaoly]-2,5-diphenyltetrazolium bromide (MTT) were purchased from Sigma-Aldrich (St. Louis, MO, USA). Docosahexaenoic acid (DHA) was obtained from Cayman Chemical (Ann Arbor, MI, USA). Human recombinant stem cell factor (SCF) protein was purchased from R&D Systems (Minneapolis, MN, USA). Primary antibodies used in this study were obtained from Santa Cruz Biotechnology. (Santa Cruz, CA, USA): γ-GCS (sc-55586) and β-actin (sc-1616) antibodies. Primary antibodies including anti-phosphorylation-Akt (T308) (p-Akt; T308; #4060), anti-phosphorylation-Akt (S473) (p-Akt; S473; #13038), anti-Akt (Akt; # 2964), anti-phosphorylation-eNOS (S1177) (p-eNOS; S1177; #9571), anti-eNOS (eNOS; #5880S), anti-phosphorylation-ERK 1/2 (T202/Y204) (p-ERK 1/2; T202/Y204; #9101), and anti-ERK 1/2 (ERK 1/2; #9102) were acquired from Cell Signaling Technology (Danvers, MA, USA). The commercial nuclear-cytoplasmic protein extraction kit was purchased from Pierce Biotechnology (Lackford, IL, USA). Fetal bovine serum (FBS) used in cell culture media was acquired from Thermo Fisher Scientific (Pittsburgh, PA, USA). The commercial growth kit (EGM-2 bullet kit) was obtained from Lonza (Allendale, NJ, USA). Lipofectamine LTX with plus reagent and Trizol reagent were obtained from Invitrogen (Carlsbad, CA, USA). A commercial real-time polymerase chain reaction (RT-PCR) kit was acquired from Promega Inc. (Madison, WI, USA). Specific Taqman^®^ MicroRNA assay kits, including the primers for has-miR-221 and control U6 snRNA, were obtained from Applied Biosystems (Carlsbad, CA, USA). The control plasmid vector and anti-miR-221 plasmids were obtained from System Biosciences Inc. (Mountain View, CA, USA).

### 2.2. Preparation, Characterization and Cell Culture of hEPCs

The hEPCs were prepared from human umbilical cord blood mononuclear cells (MNCs) according to the previous methods [26,27]. All protocols followed the ethical guidelines and were approved by the reviewing committee board at the institution. In brief, these MNCs were separated by adopting a previous protocol [26]. Those desired hEPCs colonies were selected and cultured in gelatin-coating tissue culture dishes in culture media with FBS and a commercial growth kit (EGM-2 kit).

Analysis of immunophenotype of hEPCs was performed by using a fluoresence-labelled cell sorting methods in a flow cytometry system according to a previous study [27]. The identification of biomarkers (CD31^+^/CD105^+^/CD144^+^/CD309^+^) was executed according to a previous protocol [27]. The identification rates of positive markers were above 90% in gated cells.

The passages of hEPCs used in the current study were between passages 6 and 9. hEPCs were cultured in a gelatin (50 μg/mL) coating culture dish in the presence of the EGM-2 growth kit and 10% FBS MCDB-131 medium without antibiotics. TMAO was dissolved in the organic solvent dimethyl sulfoxide (DMSO).

### 2.3. Neovasculogenesis Assay

The hEPCs were cultured in Matrigel-coated 96-well plates with MCDB-131 medium in the presence or absence of SCF (20 ng/mL) or TMAO (50, 100, 150, and 300 μM) for 8 h. For the neovasculogenesis assay, 4 mg/mL Matrigel (50 μL) was added to the bottom of each well at 37 °C until gelatinization. At the end of the vascular tube formation experiment, cells were treated with glutaraldehyde/paraformaldehyde solutions and fluorescence staining solutions (calcein-AM solution). The formation of vascular tubes on Matrigels was observed and documented under inverted phase-contrast microscopy (Olympus IX-71 model) at 40X, and digital photos were analyzed with the Olympus digital imaging system (DP-71 system; Olympus, Tokyo, Japan).

### 2.4. Assessment of Cell Proliferation

Cell proliferation levels were measured using the MTT assay, following a previously established protocol [6]. The hEPCs were seeded at a density of 2 × 10^4^ cells per well in a 24-well plate and cultured in media containing SCF (20 ng/mL) in the presence or absence of TMAO at concentrations of 50, 100, 150, and 300 μM. The experiments were performed in triplicate and repeated at least three times. At the end of the experiments, cultured media were removed from each plate, and 0.5 mg/mL MTT reagent was added to each well. After incubation in a cell culture incubator, an isopropanol solvent was added to each well after the removal of the MTT solution. All culture plates were vibrated to dissolve the MTT crystals. Optical density was measured using a multi-well plate reader at a wavelength of 570 nm.

### 2.5. Extraction of Cellular Proteins and Western Blotting Analysis

Protein extractions were performed using a commercial kit in the presence of inhibitors of phosphatase and proteinase. In order to separate nuclear and cytoplasmic fractions, the cellular extraction solution was centrifuged at 12,000× *g* for 10 min. The remaining supernatant was collected as a cytoplasmic protein part. Cytoplasmic proteins (50 μg) were fractioned by using 10% sodium-dodecyl sulfate polyacrylamide gel electrophoresis (SDS-PAGE). The resulting SDS-PAGE gel was blotted onto a polyvinylidene difluoride (PVDF) membrane and detected with a primary monoclonal antibody against the γ-GCS protein. These blots were stripped and reprobed with an internal control antibody against β-actin. The measurements of other targeted proteins such as p-Akt, p-eNOS, and p-ERK1/2 were executed using the procedure described above. These blots were stripped and reprobed with Akt, eNOS, or ERK1/2 primary antibodies and used as internal controls, respectively.

### 2.6. Quantitative Real-Time PCR (qPCR)

The total RNA samples were extracted using Trizol reagent and then converted into cDNA using a commercial RT-PCR kit. These cDNA samples were then applied to a PCR reaction solution consisting of specific primers for has-miR-221. U6 snRNA was adopted as an internal control. Quantitative PCR analyses were executed using the real-time PCR system (Applied Biosystems, Carlsbad, CA, USA). Expression levels of miR-221 were normalized to the internal control U6 snRNA and presented as fold changes compared to the corresponding control subgroup. To knockdown the expression of miR-221, anti-sense plasmids against miR-221 (anti-miR-221) and an internal control vector were transfected into hEPCs using Lipofectamine LTX with plus transfection reagent according to the manufacturer’s protocol.

### 2.7. Measurement of Thiol Compounds and Metabolites

The methods for analysis of thiol compounds and metabolites were referred to in the previous protocol [28]. To measure thiol compounds, extraction samples were mixed with the reaction solution (20 mM sodium carbonate at pH 9.5, 0.2 mM ^13^C_6_-2-iodoacetaniline). These reaction mixtures were incubated at 70 °C for 2 h, and the reaction was stopped by the addition of 2% formic acid. Mixture samples were centrifuged at 14,000 rpm, and supernatants were collected for further analysis using Vion/IMS/QTOF systems.

For the measurement of metabolites, cellular extraction samples were mixed with the reaction solution (30 μL of ddH_2_O, 5 μL of 0.3 M of aniline dissolved in HCl, and 5 μL of 20 mg/mL *N*-(3-dimethylaminopropyl)-ethylcarbodiimide hydrochloride). The reaction mixture was vortexed, then centrifuged at 14,000 rpm and incubated at room temperature for 2 h. The reaction was stopped by the addition of 10% ammonium hydroxide, followed by an additional incubation at room temperature. Reaction samples were centrifuged at 14,000 rpm, and supernatants were collected for further analysis using the Vion/IMS/QTOF systems.

Briefly, we executed the analysis using liquid chromatography (LC), electrospray ionization (ESI), and mass spectrometry (MS) in this study. The LC/ESI/MS system contained an ultra-performance liquid chromatography (UPLC) system (ACQUITY UPLC I-Class, Waters) and an ESI/APCI source of a 4 kDa quadrupole time-of-flight (TOF) mass spectrometer (Waters VION, Waters). Separation of the samples was executed using the reversed-phase liquid chromatography (RPLC) technique on a BEH C18 column (2.1 × 100 mm, Walters) with sample injection at 5 μL. The elution process began with an initial flow of 99% mobile phase A (consisting of ultrapure water and 0.1% formic acid) and 1% mobile phase B (100% methanol and 0.1% formic acid), which was maintained for 0.5 min. Then, the proportion of mobile phase B was gradually increased and reached 90% in 5.5 min, followed by a 1-min hold period. The proportion of mobile phase B was then lowered to 1% within 1 min. The column was subsequently equilibrated by pumping 1% B for 4 min. The LC-ESI-MS chromatogram was obtained using the ESI+ mode with the following conditions: the capillary voltage was maintained at 2.5 kV, the source temperature was kept at 100 °C, the desolvation temperature was regulated at 250 °C, the cone gas was maintained at 10 L/h, the desolvation gas was maintained at 600 L/h, and the acquisition was carried out by the MSE mode with a scan time of 0.5 s over a range of *m*/*z* 100–1000. The data obtained was processed using the UNIFI software (Waters), which provided an illustrated chromatogram and summarized the signals’ integrated area.

### 2.8. Statistical Analysis

All experiments were executed in triplicate and repeated at least three times to confirm reproducibility. Statistical analyses of neovasculogenesis, migration, miR-221, GSH levels and GSH/GSSG ratio were performed to determine the difference among subgroups by SAS statistical software (Cary, NC, USA). Confirmation of statistical significance was executed by using the one-way ANOVA model and Tukey’s post hoc test at the *p* = 0.05 level. A statistical significance in protein expression between experimental and control groups was examined using the Student *t* test at the *p* = 0.05 level.

## 3. Results

### 3.1. TMAO Impaired SCF-Mediated Neovasculogenesis in hEPCs

**Figure 1:**
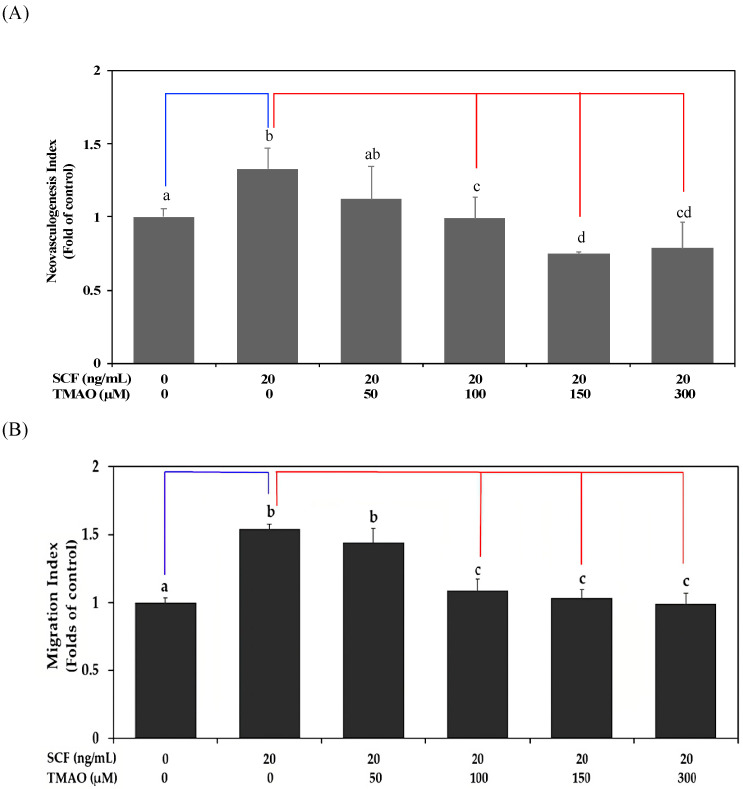
TMAO impaired SCF-mediated neovasculogenesis in hEPCs. hEPCs were stimulated with SCF protein (20 ng/mL) in the presence or absence of TMAO (50, 100, 150, and 300 μM) for 8 h. (**A**) Neovasculogenesis of hEPCs was performed according to the description in the Section 2. (**B**) Cellular migration level was analyzed according to the method described in the Section 2. The neovasculogenesis index and migration index were expressed as the mean ± SD (standard deviation). All experiments were performed in triplicate and repeated at least three times to confirm reproducibility. The analysis of biostatistical differences was performed using the one-way ANOVA model and Tukey’s post hoc test at the *p* = 0.05 level. Different letters represent statistically significant differences among different subgroups (*p* < 0.05). A blue line represents a statistically significant difference in comparison with the control subgroup (*p* < 0.05). A red line represents statistically significant differences in comparison with the SCF alone-treated subgroup (*p* < 0.05).

The SCF plays an important role in the induction of neovasculogenic activities in EPCs [5]. However, TMAO is a risk factor for atherosclerosis and CVD [15]. In this study, we investigated whether TMAO could affect SCF-mediated neovascularization in hEPCs. As shown in Figure 1A,B, treatment with recombinant human SCF protein significantly induced neovascularization (Figure 1A) and cell migration (Figure 1B) in hEPCs in vitro (*p* < 0.05). However, treatment with TMAO dose-dependently impaired neovasculogenesis and cell migration levels in hEPCs upon stimulation of SCF protein (*p* < 0.05). The results indicated that TMAO markedly impaired hEPCs function and inhibited SCF-mediated neovascularization and migration capabilities.

### 3.2. TMAO Blocked SCF-Mediated Activation of Akt/eNOS and MAPK/ERK Signaling Pathways in hEPCs

**Figure 2:**
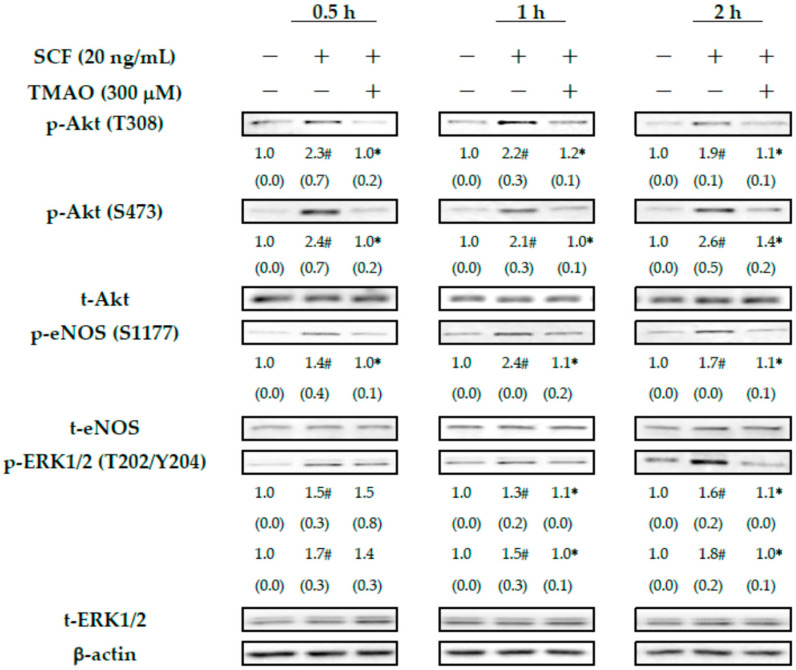
TMAO blocked SCF-mediated activation of Akt/eNOS and MAPK/ERK signaling pathways in hEPCs. hEPCs were stimulated with SCF protein (20 ng/mL) in the presence or absence of TMAO (300 μM) for different time points (0.5, 1, and 2 h). Measurement of cytoplasmic proteins was performed using antibodies against p-Akt (T308), p-Akt (S473), p-eNOS (S1177), p-ERK1/2 (T202/Y204), t-Akt, t-eNOS, t-ERK1/2, and actin by using Western blotting analysis as described in the Section 2. The expression level (integrated densities) of each protein was presented as the mean ± standard deviation (SD). The mean values of integrated densities were adjusted with the corresponding control proteins (t-Akt, t-eNOS, and t-ERK1/2) and shown in the bottom row. The values of SD were presented in parenthesis. A biostatistically significant difference in protein expression level between experimental and control subgroups was examined using the Student *t* test at the *p* = 0.05 level. A pound sign (#) represented a statistically significant difference in comparison with the untreated-control subgroup (*p* < 0.05). An asterisk sign (*) represented a statistically significant difference in comparison with the SCF alone-treated subgroup (*p* < 0.05).

Our previous study demonstrated that Akt/eNOS signaling pathways play important roles in neovasculogenesis [6]. Our results already showed that TMAO could suppress SCF-mediated neovasculogenesis in hEPCs (Figure 1). Therefore, we investigate whether TMAO could modulate Akt/eNOS signaling cascades in hEPCs upon stimulation of SCF protein. Treatment of SCF protein effectively induced the phosphorylation of Akt and eNOS signaling proteins in hEPCs at different time points (0.5, 1, and 2 h) (Figure 2). Treatment with SCF protein further induced the phosphorylation of ERK1/2 proteins in hEPCs. However, treatment with TMAO blocked the action of the SCF protein and inhibited the phosphorylation levels of Akt, eNOS, and ERK 1/2 signaling proteins in hEPCs. These results suggested that treatment of SCF protein significantly induced the phosphorylation of Akt, eNOS, and ERK1/2 signaling molecules in hEPCs. However, treatment of TMAO could impair SCF-mediated neovasculogenesis in association with the inactivation of Akt/eNOS and MAPK/ERK signaling pathways in hEPCs.

### 3.3. TMAO Inhibited SCF-Mediated Neovasculogenesis through Upregulation of miR-221 in hEPCs

**Figure 3:**
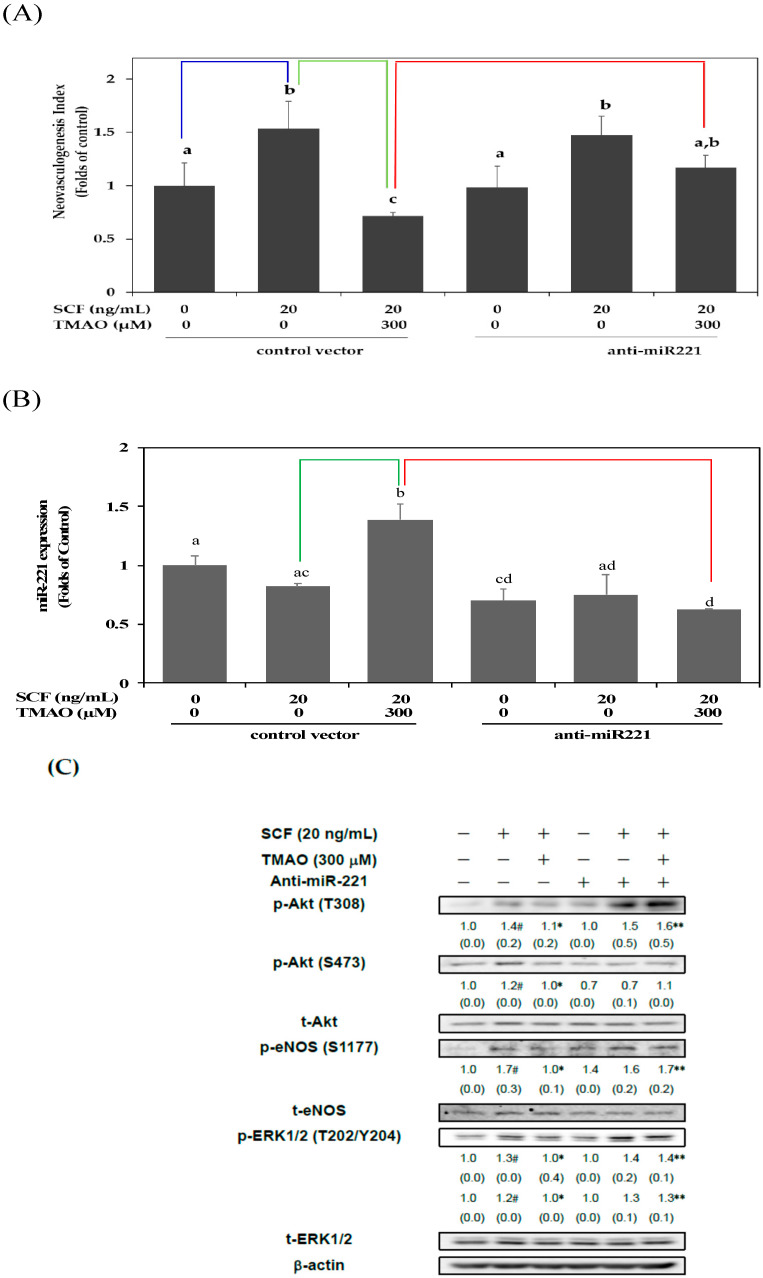
TMAO inhibited SCF-mediated neovasculogenesis through upregulation of miR-221 in hEPCs. hEPCs were transfected with a control vector or anti-sense plasmid against miR-221 (anti-miR-221) for 24 h. (**A**) At the end of plasmid transfection, hEPCs were treated with SCF protein (20 ng/mL) in the presence or absence of TMAO (300 μM) for 8 h. A detailed neovasculogenesis procedure was described in the Section 2. The analysis of biostatistical differences was performed using the one-way ANOVA model and Tukey’s post hoc test at the *p* = 0.05 level. Different letters represented statistically significant differences among different subgroups (*p* < 0.05). A blue line represents a statistically significant difference in comparison with the control subgroup (*p* < 0.05). A green line represents a statistically significant difference in comparison with the SCF alone-treated subgroup (*p* < 0.05). A red line represents a statistically significant difference in comparison with cotreatment of SCF and TMAO subgroup (*p* < 0.05). (**B**) At the end of plasmid transfection, hEPCs were treated with SCF protein (20 ng/mL) in the presence or absence of TMAO (300 μM) for 8 h. Measurement of miR-221 was performed using qPCR analysis as described in Materials and Methods. The analysis of biostatistical differences was performed using the one-way ANOVA model and Tukey’s post hoc test at the *p* = 0.05 level. Different letters represented statistically significant differences among different subgroups (*p* < 0.05). A green line represents a statistically significant difference in comparison with the SCF alone-treated subgroup (*p* < 0.05). A red line represents a statistically significant difference in comparison with cotreatment of SCF and TMAO subgroup (*p* < 0.05). (**C**) At the end of plasmid transfection, hEPCs were cultured with SCF (20 ng/mL) in the presence or absence of TMAO (300 μM) for 8 h. Measurements of cytoplasmic proteins were performed using antibodies against p-Akt (T308/S473), p-eNOS (S1177), p-ERK1/2 (T202/Y204), t-Akt, t-eNOS, t-ERK1/2, and β-actin by using Western blotting analysis as described in Materials and Methods. The mean values of integrated densities were adjusted with the corresponding control proteins. The values are presented as mean ± standard deviation (SD). The mean values of integrated densities are shown in the bottom row. The values of SD were presented in parenthesis. A biostatistically significant difference in protein expression level between experimental and control subgroups was examined using the Student *t* test at the *p* = 0.05 level. A pound sign (#) represented a statistically significant difference in comparison with the untreated-control subgroup (*p* < 0.05). An asterisk (*) represents a statistically significant difference in comparison with the SCF alone-treated subgroup (*p* < 0.05). A double asterisk sign (**) represented a statistically significant difference in comparison with cotreatment of SCF and TMAO subgroup (*p* < 0.05).

Our previous studies suggested that miR-221 was involved in the dysregulation of neovasculogenesis in hEPCs [6,19]. Therefore, we further investigated whether TMAO could inhibit SCF-mediated neovascularization through the modulation of miR-221 expression in hEPCs. As shown in Figure 3A, transfection of an anti-sense plasmid against miR-221 (anti-miR-221) could reverse TMAO-mediated impairment of neovasculogenesis in hEPCs upon stimulation of SCF. It was suggested that TMAO-mediated suppression of neovasculogenesis, in part, was associated with an upregulation of miR-221 in hEPCs. Therefore, we further measured the expression of miR-221 in hEPCs upon stimulation with TMAO. As shown in Figure 3B, treatment with TMAO significantly induced the expression of miR-221 in hEPCs even in the presence of SCF. The expression of miR-221 in hEPCs was significantly reduced upon transfection with an anti-miR-221 plasmid.

In order to verify these important findings, we investigated whether TMAO could inhibit the activation of Akt/eNOS and ERK1/2 signaling pathways through an upregulation of miR-221 in hEPCs upon the stimulation of SCF. As shown in Figure 3C, transfection of an anti-miR-221 plasmid could enhance the phosphorylation of Akt, eNOS, and ERK1/2 signaling proteins in hEPCs in the presence of TMAO. These results suggested that TMAO-mediated expression of miR-221 played an important role in the inactivation of Akt/eNOS and ERK1/2 signaling pathways in hEPCs upon SCF protein stimulation.

### 3.4. DHA Alleviated TMAO-Mediated Suppression of Neovasculogenesis in Association with a Decreased Level of miR-221 in hEPCs

Our previous study demonstrated the protective effects of DHA on the dysfunction of hEPCs [25]. The above results already indicated that TMAO could induce the expression of miR-221 and inactivate neovasculogenic signaling pathways including Akt, eNOS, and ERK 1/2 proteins in hEPCs (Figure 3C). Therefore, we further investigated whether DHA could protect hEPCs from TMAO-induced cell dysfunction.

**Figure 4:**
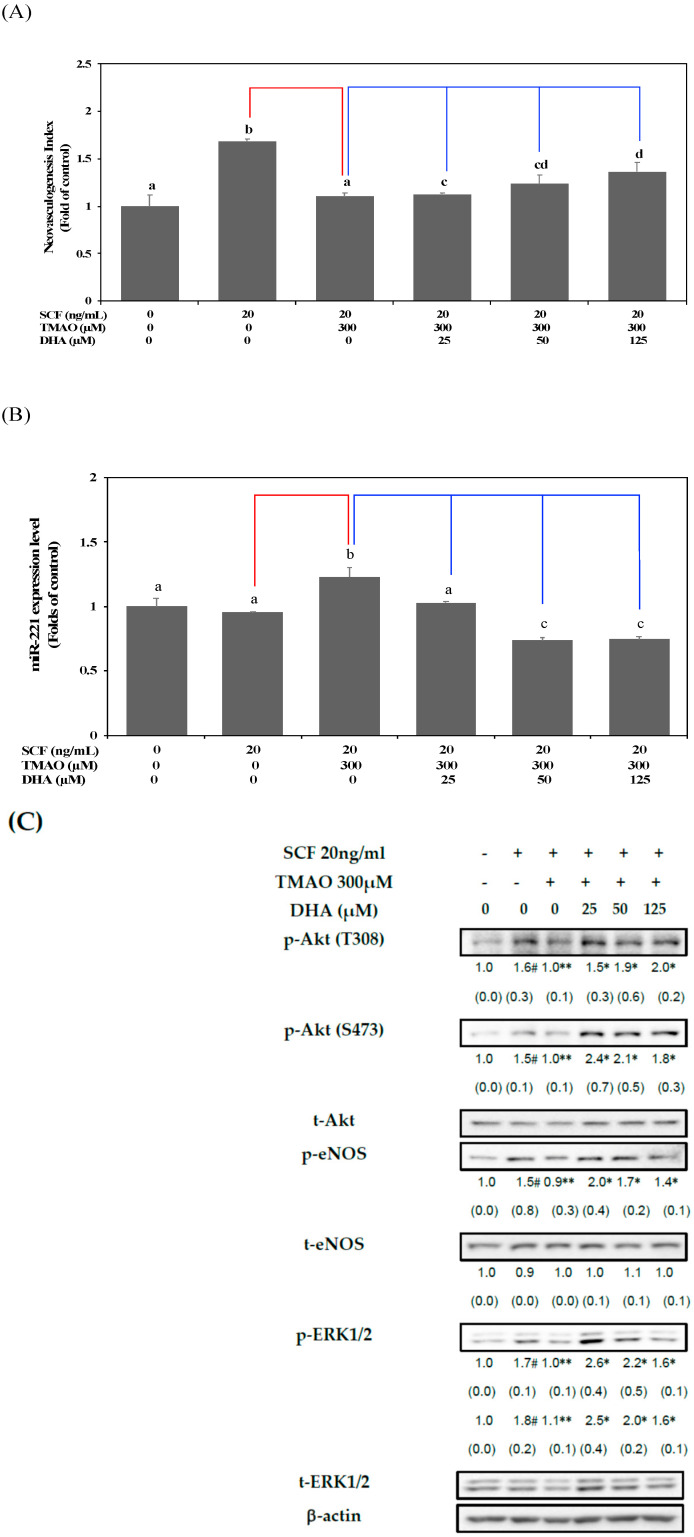
DHA alleviated TMAO-mediated suppression of neovasculogenesis in association with a decreased level of miR-221 in hEPCs. hEPCs were cultured with SCF (20 ng/mL) and TMAO (300 μM) in the presence of DHA (25, 50, and 125 μM). (**A**) Neovasculogenesis (at the 8 h time point) of hEPCs was performed according to the description in the Section 2. The analysis of biostatistical differences was performed using the one-way ANOVA model and Tukey’s post hoc test at the *p* = 0.05 level. Different letters represented statistically significant differences among different subgroups (*p* < 0.05). (**B**) Measurement of miR-221 (at the 8 h time point) was performed by using qPCR analysis as described in Materials and Methods. The analysis of biostatistical differences was performed using the one-way ANOVA model and Tukey’s post hoc test at the *p* = 0.05 level. Different letters represent statistically significant differences among different subgroups (*p* < 0.05). (**C**) Measurements of cytoplasmic proteins (at the 2 h time point) were performed using antibodies against p-Akt (T308/S473), p-eNOS (S1177), p-ERK1/2 (T202/Y204), t-Akt, t-eNOS, t-ERK1/2, and actin by using Western blotting analysis as described in Materials and Methods. The mean values of integrated densities were adjusted with the corresponding control proteins (t-Akt, t-eNOS, and t-ERK1/2). The values are presented as mean ± SD. The mean values of integrated densities are shown in the bottom row. The values of SD were presented in parenthesis. A biostatistically significant difference in protein expression level between experimental and control subgroups was examined using the Student *t* test at the *p* = 0.05 level. A pound sign (#) represented a statistically significant difference in comparison with the untreated-control subgroup (*p* < 0.05). A double asterisk sign (**) represented a statistically significant difference in comparison with the SCF alone-treated subgroup (*p* < 0.05). An asterisk sign (*) represented a statistically significant difference in comparison with cotreatment of SCF and TMAO-treated subgroups (*p* < 0.05). A red line represents a statistically significant difference in comparison with the SCF alone-treated subgroup (*p* < 0.05). A blue line represents statistically significant differences in comparison with cotreatment of SCF and TMAO subgroup (*p* < 0.05).

The treatment with DHA dose-dependently reversed the neovascularization level in hEPCs upon TMAO stimulation (Figure 4A). Moreover, the expression of miR-221 in hEPCs was significantly inhibited by DHA in the presence of TMAO (*p* < 0.05) (Figure 4B). To further verify these findings, we investigated whether DHA could reverse TMAO-mediated inactivation of Akt/eNOS and MAPK/ERK signaling pathways in hEPCs. Treatment with DHA resulted in an increase in the phosphorylation levels of Akt, eNOS, and ERK 1/2 proteins in hEPCs upon stimulation of TMAO (Figure 4C).

These results suggested that DHA could block the action of TMAO and enhance neovasculogenesis through downregulation of miR-221 and activation of the Akt/eNOS and MAPK/ERK signaling pathways in hEPCs.

### 3.5. DHA Alleviated TMAO-Mediated Suppression of Neovasculogenesis in Association with an Increased GSH Level and GSH/GSSG Ratio in hEPCs

**Figure 5:**
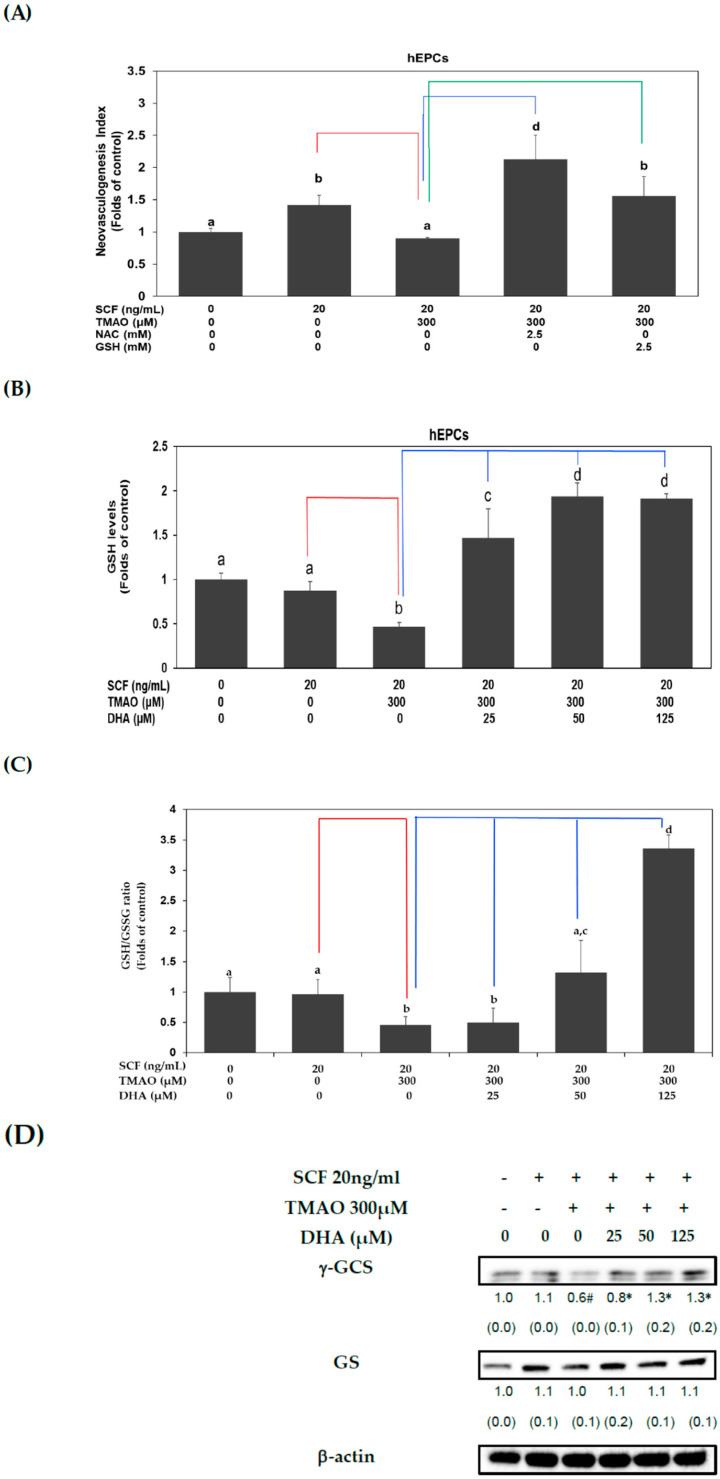
DHA alleviated TMAO-mediated suppression of neovasculogenesis in association with an increased GSH level and GSH/GSSG ratio in hEPCs. (**A**) hEPCs were cultured with SCF (20 ng/mL) and TMAO (300 μM) in the presence of NAC or GSH (2.5 mM) for 8 h. Neovasculogenesis of hEPCs was performed according to the description in the Section 2. The analysis of biostatistical differences was performed using the one-way ANOVA model and Tukey’s post hoc test at the *p* = 0.05 level. Different letters represent statistically significant differences among different subgroups (*p* < 0.05). A red line represents a statistically significant difference in comparison with the SCF alone-treated subgroup (*p* < 0.05). A blue or green line represents a statistically significant difference in comparison with cotreatment of SCF and TMAO subgroup, respectively (*p* < 0.05). Measurements of cellular GSH level (**B**) and GSH/GSSG ratio (**C**) (at the 8 h time point) were performed using LC/ESI/MS as described in Materials and Methods. The analysis of biostatistical differences was performed using the one-way ANOVA model and Tukey’s post hoc test at the *p* = 0.05 level. Different letters represent statistically significant differences among different subgroups (*p* < 0.05). A red line represents a statistically significant difference in comparison with the SCF alone-treated subgroup (*p* < 0.05). A blue line represents statistically significant differences in comparison with cotreatment of SCF and TMAO subgroup (*p* < 0.05). (**D**) Measurements of cytoplasmic proteins (at the 2 h time point) were performed using antibodies against γ-GCS and GS proteins by using Western blotting analysis as described in Materials and Methods. The mean values of integrated densities were adjusted with the internal control β-actin proteins. The values are presented as mean ± SD. The mean values of integrated densities were shown in the bottom row. The values of SD are presented in parenthesis. A biostatistically significant difference in protein expression level between experimental and control subgroups was examined using the Student *t* test at the *p* = 0.05 level. A pound sign (#) represented a statistically significant difference in comparison with the SCF alone-treated subgroup (*p* < 0.05). An asterisk sign (*) represented a statistically significant difference in comparison with cotreatment of SCF and TMAO-treated subgroups (*p* < 0.05).

Previous studies indicated that TMAO could increase oxidative stress in ECs [29,30]. It is plausible that TMAO could act as an oxidant and promote the dysfunction of hEPCs. Thus, we conducted further investigation to determine whether TMAO could impair neovasculogenesis by elevating oxidative stress levels in hEPCs. Treatment with antioxidants such as *N*-acetylcysteine (NAC) or a reduced form of glutathione (GSH) could significantly reverse TMAO-mediated suppression of neovasculogenesis in hEPCs, respectively (*p* < 0.05) (Figure 5A). Therefore, we further studied whether DHA could regulate GSH levels in hEPCs upon stimulation of SCF protein and TMAO. As shown in Figure 5B,C, treatment with TMAO significantly suppressed intracellular GSH levels and the GSH/GSSG ratio in hEPCs upon stimulation of SCF protein (*p* < 0.05). However, treatment with DHA significantly increased cellular GSH levels (Figure 5B) and the GSH/GSSG ratio (Figure 5C) in hEPCs, respectively (*p* < 0.05). These results demonstrated that DHA could reverse TMAO-mediated suppression of neovasculogenesis, in part, through an increment in cellular GSH level and GSH/GSSG ratio in hEPCs. It is well known that GSH is synthesized sequentially by several enzymes, such as γ-GCS and GSH synthetase (GS). Therefore, we further examined whether DHA could regulate the protein expression levels of γ-GCS and GS in hEPCs upon TMAO stimulation. Our results showed that treatment with TMAO could suppress the expression of γ-GCS but not GS protein in hEPCs (Figure 5D). Importantly, DHA significantly augmented the expression of γ-GCS rather than the GS protein in hEPCs. Our results indicated that DHA could enhance the expression of γ-GCS protein and increase cellular GSH levels upon TMAO stimulation in hEPCs.

## 4. Discussion

Previous studies showed that plasma levels of TMAO are highly correlated with the prognosis of peripheral arterial disease and the risk of CVD [31,32,33]. SCF protein could modulate neovasculogenesis in hEPCs and lower the risk of CVD [5]. In experimental animals, increasing levels of TMAO could enhance oxidative stress and impair neovasculogenesis [33]. A previous study suggested that consumption of fish oil abundant with N-3 PUFAs such as DHA and EPA could reduce plasma levels of TMAO through an increased renal clearance rate [34]. In this study, our results demonstrated that SCF could enhance the neovascularization and cellular movement capabilities of hEPCs in vitro (Figure 1). TMAO significantly impaired SCF-mediated neovasculogenesis and migration in hEPCs (Figure 1). The inhibitory effects of TMAO on SCF-stimulated hEPCs were through an inactivation of Akt, eNOS, and ERK1/2 signaling proteins in hEPCs (Figure 2). These findings suggested that TMAO could induce dysfunction of hEPCs through an impairment of SCF-mediated signaling pathways, including Akt/eNOS and MAPK/ERK cascades. These results were consistent with other findings and suggested important roles for Akt/eNOS signaling molecules in neovasculogenesis in hEPCs [5,6]. SCF protein could induce the activation of the Akt molecule (p-Akt T308/p-Akt S473) and ERK1/2 (p-ERK1/2 T202/Y204) at an early stage (0.5 h) as well as the eNOS molecule (p-eNOS S1177) at a later stage (2 h) (Figure 2). However, treatment with TMAO could significantly inhibit the phosphorylation levels of Akt, eNOS, and ERK1/2 signaling proteins in hEPCs (Figure 2) (*p* < 0.05).

Our previous study suggested that miR-221 plays an important role in the regulation of neovasculogenesis in hEPCs [6]. Therefore, we further investigated whether miR-221 played an important role in TMAO-mediated dysfunction of hEPCs. As shown in Figure 3, transfection of the anti-miR 221 plasmids could significantly reverse the neovasculogenesis level in hEPCs upon treatment with TMAO (Figure 3A) (*p* < 0.05). These results suggest that miR-221 plays an important role in TMAO-mediated impairment of neovasculogenesis and dysfunction of hEPCs. Our results further demonstrated that treatment with TMAO could significantly upregulate the expression of miR-221 in hEPCs (Figure 3B) (*p* < 0.05). We further investigate the regulatory effects of the anti-miR 221 plasmids on key signaling proteins such as Akt, eNOS, and ERK1/2. As shown in Figure 3C, transfection of an anti-miR 221 plasmid could further inhibit the phosphorylation (activation) of neovasculogenesis signaling proteins, including Akt, eNOS, and ERK1/2, in hEPCs. These results suggested that TMAO-mediated dephosphorylation and inactivation of Akt, eNOS, and ERK1/2 proteins were associated with an upregulation of miR-221 in hEPCs upon stimulation of TMAO. These results were consistent with our previous findings indicating that miR-221 played a central role in the suppression of neovascularization in hEPCs [6,35].

We further examined the beneficial effects of DHA on the prevention of TMAO-mediated dysfunction in hEPCs. As shown in Figure 4A, treatment with TMAO significantly inhibited SCF-mediated neovasculogenesis in hEPCs. In contrast, treatment with DHA dose-dependently reverses neovascularization levels in hEPCs upon stimulation of TMAO. DHA significantly inhibited the expression of miR-221 in hEPCs (Figure 4B). Moreover, treatment with DHA effectively reversed the phosphorylation (activation) of Akt, eNOS, and ERK1/2 proteins in hEPCs upon stimulation of TMAO. These results suggested that DHA could dose-dependently rescue TMAO-mediated impairment of hEPCs and prevent vascular dysfunction.

Our results further showed that TMAO inhibited SCF-mediated neovascularization through an increase in oxidative stress. Treatment with NAC or GSH could reverse TMAO-mediated suppression of neovascularization in hEPCs (Figure 5A). As shown in Figure 5B,C, treatment with TMAO significantly suppressed intracellular GSH levels and the GSH/GSSG ratio in hEPCs. However, treatment with DHA could dose-dependently reverse intracellular GSH levels and GSH/GSSG ratios in hEPCs. Moreover, DHA could increase the expression of the g-GCS protein in hEPCs (Figure 5D). These results suggested that TMAO is an oxidant and inhibits the neovasculogenic function of hEPCs.

**Figure 6:**
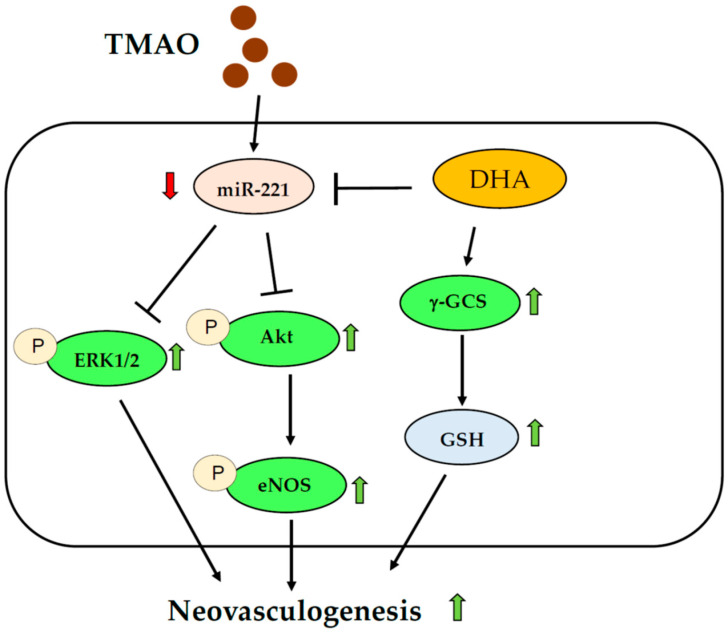
Proposed mechanism of DHA against TMAO-mediated dysfunction of hEPCs. Green arrows indicate an increased level; Red arrows indicate a decreased level.

Previous studies suggested that plasma levels of TMAO were associated with decreased function of hEPCs [15]. We provided novel evidence and a detailed mechanism of action for how TMAO might affect the activity of hEPCs (Figure 6). Interestingly, our results further demonstrated that N-3 PUFAs such as DHA could effectively block the action of TMAO to reverse the neovasculogenesis level in hEPCs. Treatment with DHA activated the Akt/eNOS and ERK1/2 signaling pathways through a downregulation of miR-221 in hEPCs. DHA could also modulate cellular levels of GSH and the GSH/GSSG ratio to rescue TMAO-mediated suppression of neovasculogenesis in hEPCs (Figure 6).

This is the first evidence showing that DHA could act as an effective nutrient to induce neovasculogenesis through an upregulation of GSH levels, activation of AKt/eNOS pathways, and downregulation of miR-221 in hEPCs.

## 5. Conclusions

In conclusion, we demonstrated a novel mechanism in which DHA could act as an effective nutrient to prevent TMAO-mediated impairment of hEPCs, in part in association with a downregulation of miR-221 and activation of Akt/eNOS and MAPK/ERK signaling pathways in hEPCs. Moreover, DHA could increase cellular levels of GSH and the GSH/GSSG ratio in hEPCs (Figure 6).

## Data Availability

All data can be found in the Supplementary Materials section.

## Supplementary Materials

The following supporting information can be downloaded at: https://www.mdpi.com/article/10.3390/nu15092190/s1, Figure S1: The raw data of Figure 2; Figure S2: The raw data of Figure 3C; Figure S3: The raw data of Figure 4C; Figure S4: The raw data of Figure 5D.
