# Supplementary material for: Docosahexaenoic Acid Alleviates Trimethylamine-N-oxide-mediated Impairment of Neovascularization in Human Endothelial Progenitor Cells
Source: Nutrients. 2023 May 4;15(9):2190. doi: 10.3390/nu15092190 (PMC10180856; doi:10.3390/nu15092190)
Supplement: Supplementary file 1 [file nutrients-15-02190-s001.zip › nutrients-2263311-supplementary.pdf]

Supporting Information

Figure S1.

	0.5h			1h			2h		
hSCF 20ng/ml	-	+	+	-	+	+	-	+	+
TMAO 300μM	-	-	+	-	-	+	-	-	+
p-Akt (T308)	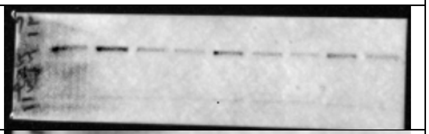								
p-Akt (T308)	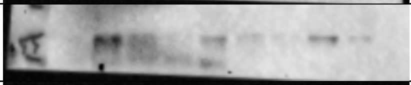								
p-Akt (S473)	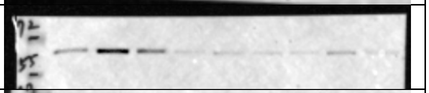								
p-Akt (S473)	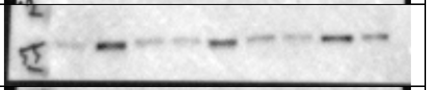								
t-Akt	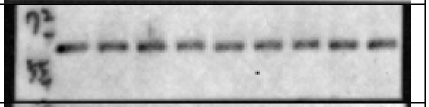								
t-Akt	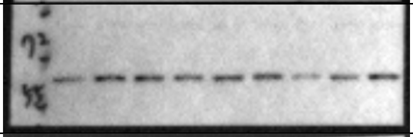								
p-eNOS (S1177)	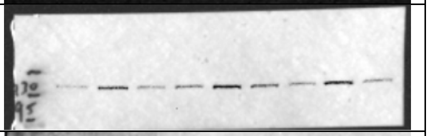								
p-eNOS (S1177)	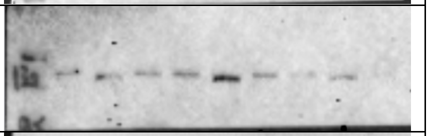								
t-eNOS	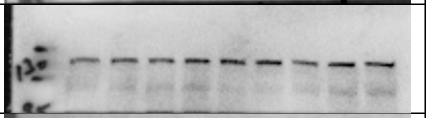								
t-eNOS	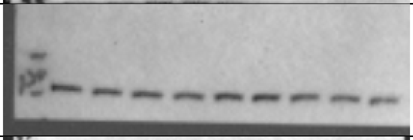								
p-ERK1/2 (T202/Y204)	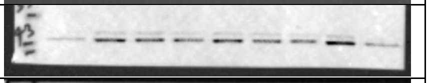								
p-ERK1/2 (T202/Y204)	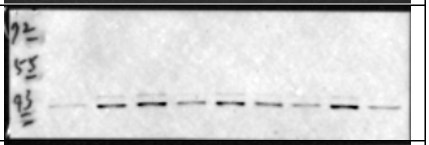								
t-ERK1/2	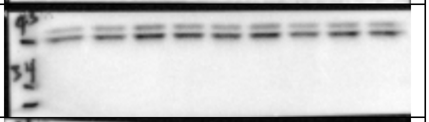								
t-ERK1/2	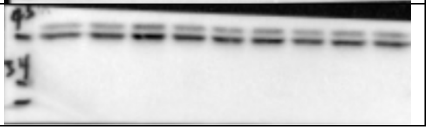								

**actin**	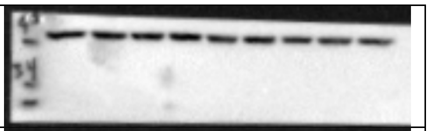
**actin**	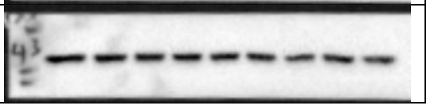

Figure S2.
(C)

	**1**						**2**					
**Control plasmid**	**+**	**+**	**+**	**-**	**-**	**-**	**+**	**+**	**+**	**-**	**-**	**-**
**Anti-miR221**	**-**	**-**	**-**	**+**	**+**	**+**	**-**	**-**	**-**	**+**	**+**	**+**
**hSCF (ng/mL)**	**0**	**20**	**20**	**0**	**20**	**20**	**0**	**20**	**20**	**0**	**20**	**20**
**TMAO (µM)**	**0**	**0**	**300**	**0**	**0**	**300**	**0**	**0**	**300**	**0**	**0**	**300**
p-Akt (T308)	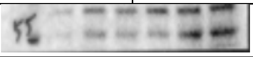						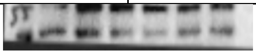					
p-Akt (S473)	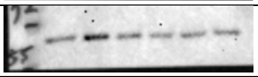						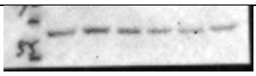					
t-Akt	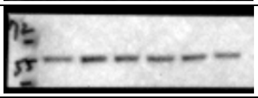						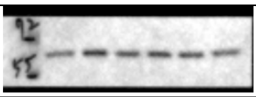					
p-eNOS	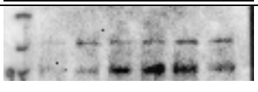						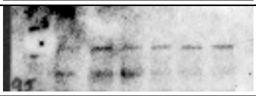					
t-eNOS	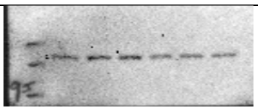						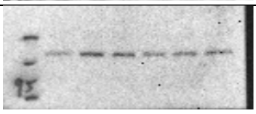					
p-ERK1/2	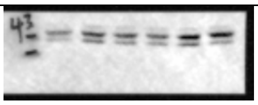						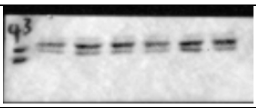					
t-ERK1/2	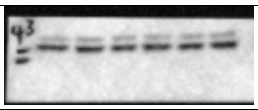						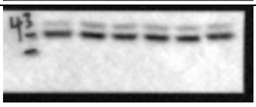					
actin	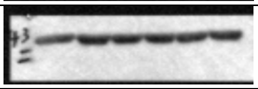						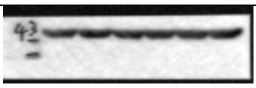					

Figure S3.

(C)

	1						2						3					
hSCF 20ng/ml	-	+	+	+	+	+	-	+	+	+	+	+	-	+	+	+	+	+
TMAO 300μM	-	-	+	+	+	+	-	-	+	+	+	+	-	-	+	+	+	+
DHA (μM)	0	0	0	25	50	125	0	0	0	25	50	125	0	0	0	25	50	125
p-Akt (T308)	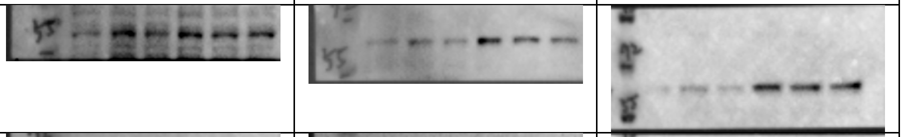																	
p-Akt (S473)	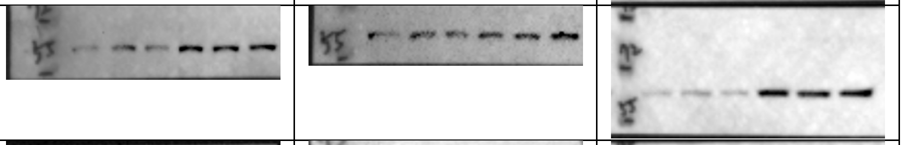																	
t-Akt	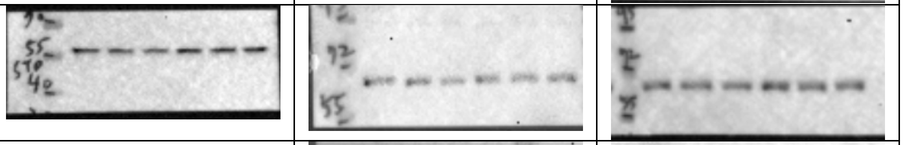																	
p-eNOS	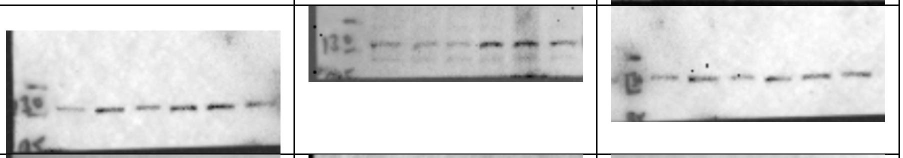																	
t-eNOS	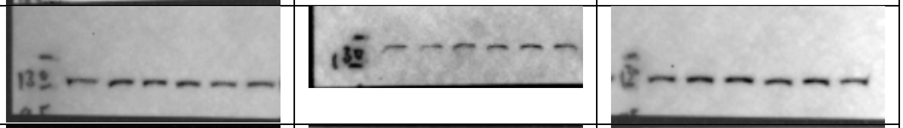																	
p-ERK1/2	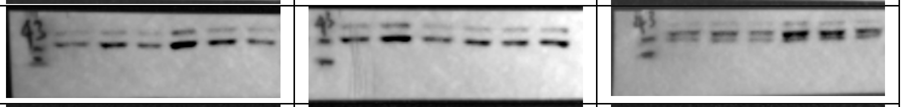																	
t-ERK1/2	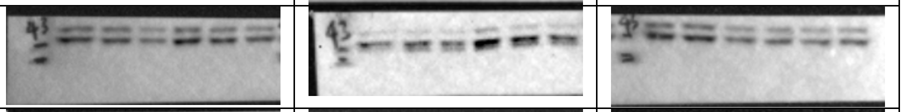																	
actin	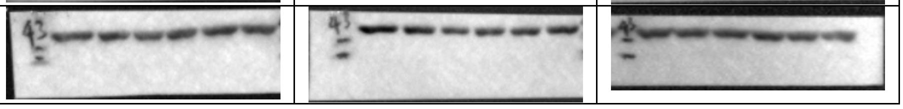																	

Figure S4.

(D)

	1	2	3
	8h	8h	8h
hSCF 20ng/ml	- + + + + +	- + + + + +	- + + + + +
TMAO 300μM	- - + + + +	- - + + + +	- - + + + +
DHA (μM)	0 0 0 25 50 125	0 0 0 25 50 125	0 0 0 25 50 125
γ-GCS	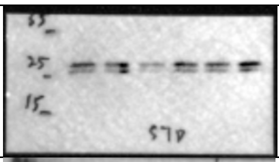	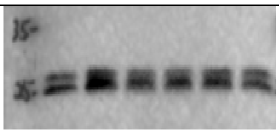	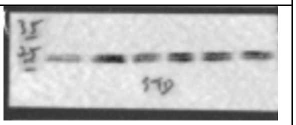
GSS	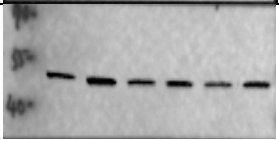	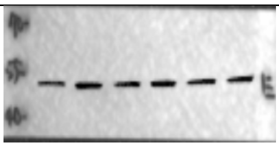	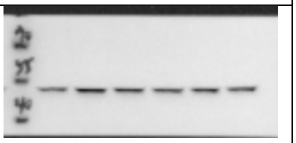
β-actin	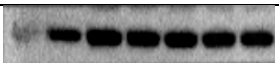	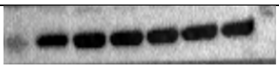	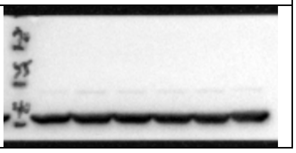
